# Dual burden of malnutrition in US Affiliated Pacific jurisdictions in the Children’s Healthy Living Program

**DOI:** 10.1186/s12889-017-4377-6

**Published:** 2017-05-22

**Authors:** Rachel Novotny, Fenfang Li, Rachael Leon Guerrero, Patricia Coleman, Aifili J. Tufa, Andrea Bersamin, Jonathan Deenik, Lynne R Wilkens

**Affiliations:** 1 University of Hawaii, 1955 East West Road, Honolulu, HI 96822 USA; 2 University of Guam, UOG Station, Mangilao, 96923 Guam; 3 Nutrition & Health Programs, Building F, PO Box 501250, Saipan, MP 96950 Northern Mariana Islands; 4 Northern Marianas College, Saipan, Northern Mariana Islands; 5 University of Alaska, AHRB, Fairbanks, AK 228 USA; 6 University of Hawaii, UH Cancer Center, Honolulu, HI USA

**Keywords:** Pacific, Race, Income, Obesity, Stunting, Country

## Abstract

**Background:**

Few data are available on dual burden of under and over nutrition of children in the Pacific region. The objective was to examine prevalence of stunting at birth and current stunting and their relationship to obesity in US Affiliated Pacific (USAP) jurisdictions.

**Methods:**

Cross sectional survey with cluster sampling by community. 5558, 2–8 years olds were measured in 51 communities in 11 USAP jurisdictions. The main outcome measures were stunting at birth, current stunting and obesity by body mass index. Prevalences of stunting at birth, current stunting and obesity were determined, adjusting for age distribution and community clustering. Differences by among age, sex, race and jurisdiction income levels were evaluated by chi-square analysis. Relationships of stunting at birth and current stunting with obesity were examined using a hierarchical model accounting for the study design.

**Results:**

Prevalences were stunting at birth 6.8% (Standard Error, SE = 0.9%), current stunting 1.4% (SE = 0.2%) and obesity 14.03.8% (SE = 0.9%). Obesity was highest in upper middle income jurisdictions (UMIJ) at 17.5%. Stunting at birth differed by race (*p* = 0.0001) with highest prevalence among Native Hawaiian/Pacific Islanders (10.7%). Prevalence of stunting at birth was different by jurisdiction income level with 27.5% in lower middle income jurisdictions (LMIJ), and 22.2% in UMIJ, and 5.5% in higher than high income jurisdictions (HIJ) at 5.5% (*p* < 0.0001). Prevalence of current stunting was higher in LMIJ than HIJ (*p* = 0.001), although children with current stunting were less likely to have been stunted at birth. The association between stunting at birth and current stunting was negative (OR = 0.19, 95% CI: 0.05–0.69).

**Conclusions:**

Currently stunted children were marginally less likely to be obese than not stunted children in the USAP, where the prevalence of current stunting is low. Stunting (at birth and current) was highest in LMIJ, while UMIJ jurisdictions had the highest dual burden of malnutrition (that is the highest combination of both stunting at birth and obesity).

**Trial registration:**

National Institutes for Health clinical trial # NCT01881373 (clinicaltrials.gov).

## Background

Stunting is the most common consequence of malnutrition worldwide, especially among low-income and middle-income countries [1, 2]. Black et al. [2] reported a global prevalence of stunting at 26% among children younger than five years old in 2011. East Africa and South-Central Asia had the highest prevalence of stunting (East Africa at 42%, and West Africa and South-Central Asia at 36%) [2]. There were substantial economic and geographic inequalities, with stunting rates samong children less than five years old 2.5 times higher in the poorest quintile of households than in the richest quintile, and 1.5 times higher in rural than in urban areas [2]. The prevalence of stunting was 28% among lower middle income countries and 7.2% among high income countries [2]. Key risk factors for stunting are maternal nutrition and infection, teenage motherhood and short birth intervals, fetal growth restriction, preterm birth, childhood nutrition and environmental factors [3]. Fetal growth restriction and preterm birth are the leading risk factors worldwide; environmental factors followed in the Pacific region.

There is epidemiological evidence demonstrating a relationship between childhood stunting and an increased risk of obesity and other adverse health consequences in adolescence and adulthood [4–6]. Sawaya et al. [7] and Hoffman et al. [8] examined possible underlying mechanisms of this relationship. They reported that childhood stunting was associated with impaired fat oxidation, a risk factor for excess weight gain when food supplies, and in particular high-fat diets, became readily available [8]. Countries in economic transition are particularly affected and show an increasing prevalence of obesity across economic levels and age groups [9, 10].

The Pacific Island countries, including the United States Affiliated Pacific (USAP) jurisdictions, are experiencing some of the highest rates of obesity in the world, in part due to substantial dietary changes that mirror changes in the regional food supply [11–13]. The shift from a high quality diet based on local foods to a nutrient-poor market diet has contributed to a decline in diet quality, nutritional status and nutrition security [12, 13]. This nutrition transition, along with other lifestyle changes such as reduced agricultural activities and a more sedentary lifestyle, are among the causes of the growing prevalence of obesity and obesity-related chronic diseases in the region, causing health officials to declare an emergency due to non-communicable (chronic) diseases [14, 15].

In the USAP, obesity prevalence is very high in the adult population [16], for example, 58% among women in the Federated States of Micronesia [16]. The USAP region is not reached by US national surveys, and prevalence data on child anthropometry are not widely available [17]. Studies have examined the prevalence of stunting and its association with obesity in Africa, South America and Asia [1–10]. A few studies were conducted in the US on “minority” groups (e.g, Hispanic), immigrants or refugee children [18, 19]. This study will examine prevalence of stunting among young children in the USAP, where predominant ethnic groups are Pacific Islander, examine stunting variability among the US Pacific populations, and examine whether stunting alters the risk for obesity. Specifically, the paper will examine, among young children in the USAP:

1. Prevalence of current stunting and stunting at birth
2. Variability in current stunting, stunting at birth, and obesity
3. The relationship between current stunting and current obesity
4. The relationship between stunting at birth and current obesity

## Methods

### Study design and sample

The Children’s Healthy Living Program for Remote Underserved Minority Populations of the Pacific Region (CHL) sample and study design has been previously described [20]. Briefly, 5972 parents and children over seven years of age provided written informed consent to participate and receive compensation prior to participation. Compensation level, determined by jurisdiction lead investigators and their IRB’s, was higher in Guam (University of Guam, $40) and Alaska (University of Alaska at Fairbanks, $50) than in Hawaii (University of Hawaii at Manoa, $20). All other jurisdictions ceded IRB approval to the University of Hawaii at Manoa (and compensation rate). Anthropometric data were collected on 5558 two to eight year old children that assented in selected communities (clusters) yielding 51 communities in 11 USAP jurisdictions in 2012 - Alaska, American Samoa, Commonwealth of the Northern Mariana Islands (CNMI), Guam, Hawaii, Federated States of Micronesia (Chuuk, Kosrae, Pohnpei,Yap), Marshall Islands, and Palau. The study was a community randomized trial that aimed to prevent childhood obesity with a multilevel multicomponent environmentally-focused intervention trial in five of the jurisdictions [Alaska, American Samoa, Commonwealth of the Northern Mariana Islands (CNMI), Guam and Hawaii], while a prevalence sample was collected in the remaining jurisdictions. As the same methods were used for the prevalence sample and the baseline sample of the intervention jurisdictions; these data were combined for this report.

### Demographic information

Child’s age was calculated based on the days elapsed between the anthropometric measurement and child’s date of birth and was categorized as 2–5 years old or 6–8 years old. Parents or caregivers reported the race and ethnicity of children by completing a questionnaire that provided check boxes according to Office of Management and Budget (OMB) categories [21] of American Indian/Alaska Native (AIAN), Asian, Black, Native Hawaiian and Pacific Islanders (NHPI), White, and More than one race group. Further detail on ethnic subgroups was gathered but is not reported here.

The USAP is characterized according to World Bank income classification [22] - Lower middle income jurisdictions (LMIJ) – Federated States of Micronesia (Chuuk, Pohnpei, Kosrae, Yap); Upper middle income Jurisdictions (UMIJ) - American Samoa, Marshall Islands, Palau; High income Jurisdictions (HIJ) - Guam, Commonwealth of the Northern Mariana Islands, US states (Hawaii and Alaska).

### Anthropometry

Child’s current height and weight were measured by trained and standardized staff [23]. Current stunting was defined as current height-for-age more than 2 SDs below the mean of the Centers for Disease Control (CDC) reference data (*n* = 5462), which are used in the region, or equivalently as current height-for-age z-score (HAZ) < −2. Extreme HAZ values were <−6 or >6 SD and were removed from the analysis. Child’s birth length was self-reported by caregivers using a questionnaire (1655 responses were given). Parents were encouraged to bring their community health cards, or medical records, to report these data whenever possible. Use of these data occurred more often in the lower middle income jurisdictions where literacy was also lower, improving integrity of these data although the percent missing was still higher in lower income jurisdictions (LMIJ = 95–99%, UMIJ = 89–99%; HIJ = 31–90%). In all jurisdictions, questionnaire data was checked on site by CHL project staff for accuracy compared to health records, and for plausibility. Stunting at birth was defined as <2 SDs below the mean of the World Health Organization day 1 reference data (*n* = 1655), which is the same reference data used by CDC for children under 2 years. Current obesity status was categorized using body mass index (kg/m^2^) based on CDC’s 2000 sex and age-specific reference data as Obese if > = 95th BMI percentile, Overweight if >85th and <95th percentile and Underweight if <5th percentile. Cutoff values for biologically implausible values defined as <−5 or >3 standard deviations (SD) for HAZ and <−4 or >5 SD for BMI z score (according to CDC reference data) were removed from the analysis.

### Statistical procedures

All analyses used survey sampling techniques [24] that weighted the sample to the young child population size in each community, using 2010 census population size of children <10 years of age for each community and that accounted for the clustering of participants in communities. The weighting resulted in representative estimates for the jurisdictions and the region, with two caveats. In Hawaii, the communities represented the non-Honolulu population and, in Alaska, the communities represented the most populous areas.

Prevalence was estimated, with 95% confidence intervals (CI), overall and stratified by sex, age group, race and jurisdiction. Prevalence was compared between groups by a chi-square test.

Logistic regression models of obesity were conducted to assess its relationship with current stunting or stunting at birth, adjusted for age group (2-5y or 6-8y), sex, and race and jurisdiction income level, with consideration for clustering and weighting as described above. A logistic model of current stunting was conducted to assess its relationship with stunting at birth. Odds ratios and 95% CI were the primary statistics reported from the models. Interactions are tested by the Wald test of cross-product terms.

Underweight and overweight children were excluded from the obesity models, and obese children were compared to healthy weight children. *P*-values <0.05 were considered statistically significant. Statistical analyses were done using Statistical Analysis Software (SAS Institute Inc., Cary, NC).

## Results

### Description of the study population

A total of 5462 (out of 5972 consented) children with anthropometric measures on current stunting status were included in the study, of which 66% were 2–5 years old and 51% were male. Almost two-thirds (61%) were NHPI, 20% were more than one race, 9% were Asian, 8% were White, 2% were AIAN and 0.3% were Black. About two-thirds of children (61%) were from HIJ; a quarter from UMIJ, and 14% were from LMIJ (Table 1).

**Table 1:** Characteristics of study participants with anthropometric measurements in the Children’s Healthy Living Program (*n* = 5462)

Child characteristics	Number	Percent^a^
Sex		
Male	2792	51.1
Female	2670	48.9
Age		
2–5 years old	3586	65.6
6–8 years old	1876	34.4
Race according to Office of Management and Budget (*n* = 5443)		
American Indian/Alaska Native	121	2.2
Asian	488	9.0
Black	16	0.3
Native Hawaiian/Pacific Islander	3323	61.1
White	415	7.6
More than one race	1080	19.8
Jurisdiction income level		
Lower middle income	747	13.7
Upper middle income	1368	25.0
High income	3347	61.3

^a^Total percent may not add up to 100 due to rounding

### Prevalences

**Fig. 1:**
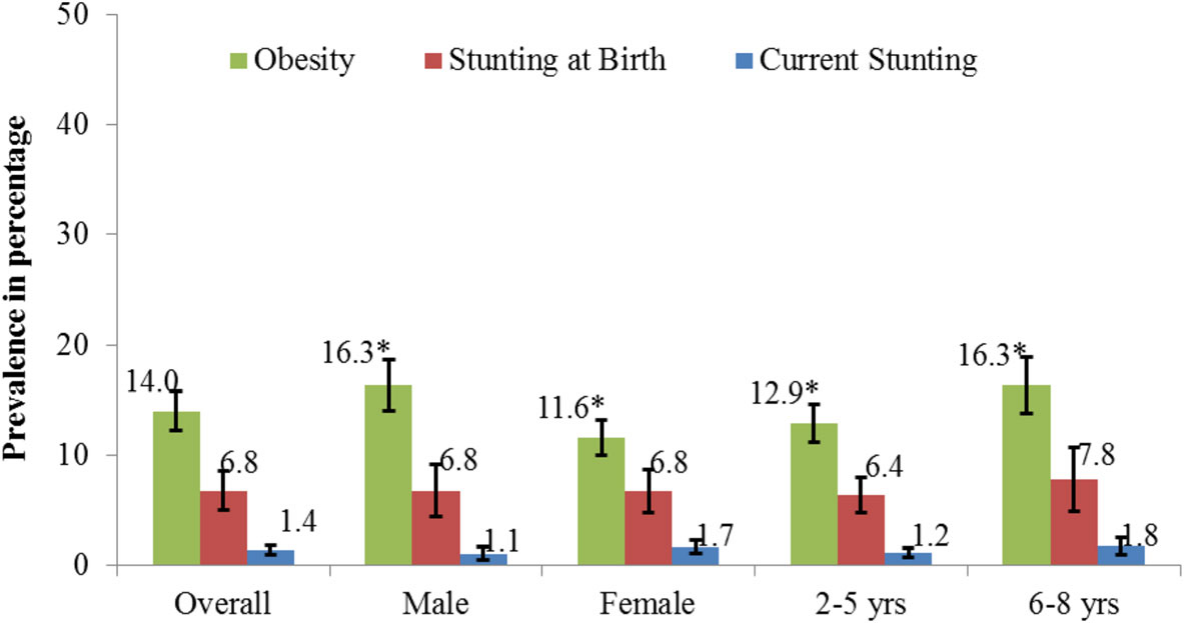
Prevalence of Obesity and Stunting, Overall and by Sex and Age. *Differences in prevalence by demographic group, *p* < 0.05 Chi-square; Error bars refer to 95% Confidence Interval of the prevalence estimate

The overall regional prevalence of young child obesity was 14.0%, stunting at birth was 6.8% and current stunting was 1.4%. Males had a higher prevalence of obesity than females (*p* < 0.001) and older children had a higher prevalence of obesity than younger children (*p* < 0.01, Fig. 1). There was no difference in prevalence of current stunting or stunting at birth by sex or age group. There was a negative association between stunting at birth and current stunting. Children who were stunted at birth were 80% less likely to be currently stunted than those who were not stunted at birth (*OR* = 0.19, 95% *CI*: 0.05–0.69).

**Fig. 2:**
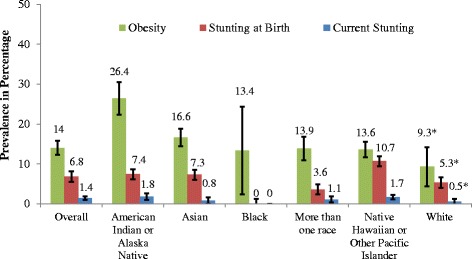
Prevalence of Obesity and Stunting by Race. *Differences in prevalence among race groups, compared to Asian, *p* < 0.05 Chi-square; Error bars refer to 95% Confidence Interval of the prevalence estimate

Prevalence of obesity, stunting at birth and current stunting varied significantly by race (*p* < 0.001, Fig. 2) with the highest levels for obesity and current stunting among AIAN (26.4% and 1.8%, respectively). Among race groups, the highest prevalence of stunting at birth was among NHPI (10.7%) whose obesity prevalence was 13.6%, showing the highest double burden of malnutrition within a race group.

**Fig. 3:**
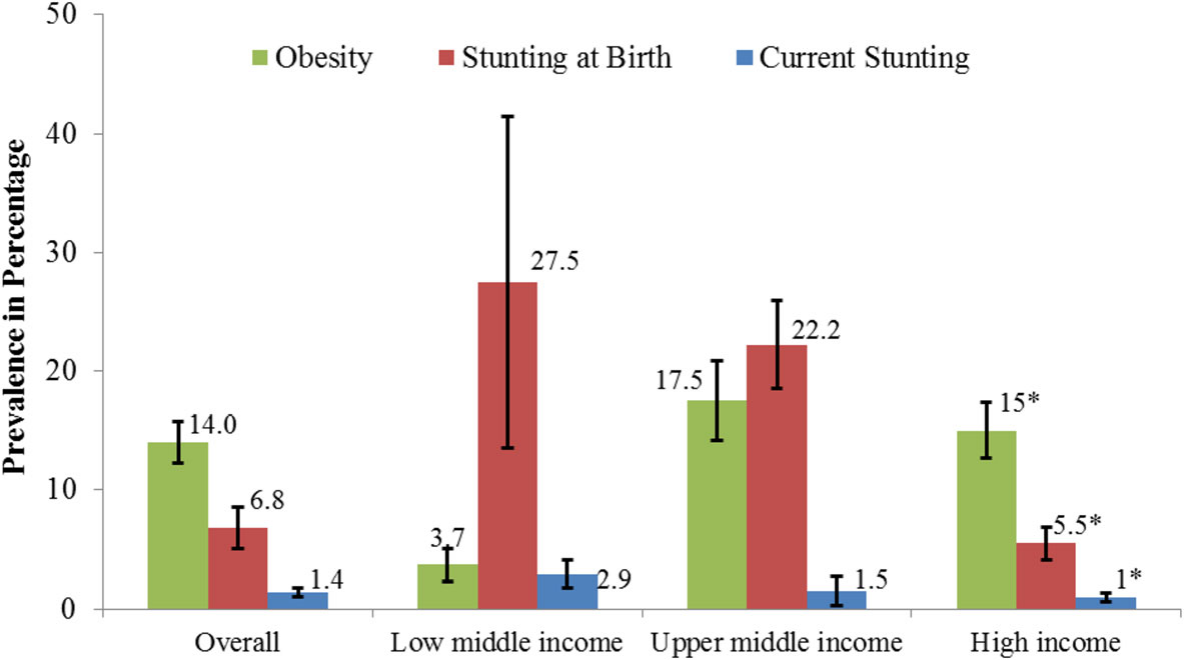
Prevalence of Stunting and Obesity by Jurisdiction Income Level. *Differences among jurisdiction income levels, *p* < 0.05, Chi-square; Error bars refer to 95% Confidence Interval of the prevalence estimate

All three anthropometric measures differed significantly by jurisdiction income level (Fig. 3). Stunting decreased as income level increased, while obesity increased sharply from LMIJ to UMIJ (3.7% to 17.5%) and then decreased slightly from UMIJ to HIJ (17.5% to 14.9%). Thus, by income level, the highest double burden of malnutrition was in UMIJ.

### Relationship between covariates and obesity

A core logistic model was fit for obesity compared to healthy weight, including the following covariates: age, sex, race, and jurisdiction income level. Children 6–8 years old were more likely than children 2–5 years old to be obese (adjusted OR = 1.28, 95% CI: 1.06–1.54, *P* = 0.01), and boys were more likely than girls to be obese (OR = 1.50, 95% CI: 1.24–1.79, *P* < 0.0001). Compared to Whites, all non-White groups were more likely to be obese. The ORs for NHPI (OR = 1.69, 95% CI: 0.93–3.08, *P* = 0.09) and More than One Race (OR = 1.61, 95% CI: 0.84–3.08, *P* = 0.15) were not significant, while the ORs for the other groups were significant: AIAN (OR = 3.86, 95% CI: 1.99–7.47, *P* < 0.0001), Asians (OR = 1.94, 95% CI: 1.09–3.47, P < =0.02), and Blacks (OR = 3.40, 95% CI: 1.42–8.12, *P* = 0.006). Children from LMIJ were less likely to be obese than those from HIJ, those (*OR* = 0.20, 95% *CI*: 0.12–0.33, *p* < 0.0001) while those from UMIJ were similar (*OR* = 1.22, 95% *CI*: 0.86–1.73, *p* = 0.27).

### Relationship between stunting and obesity

Stunting at birth was not related to current obesity (adjusted *OR* = 0.70, 95% *CI* = 0.34–1.43, *p* = 0.33).

## Discussion

In this study, we observed a low rate of both current stunting and stunting at birth. This finding was in alignment with the declines in stunting reported elsewhere in the world. For example, in Brazil, stunting declined from 37% in 1974–1975 to 7% in 2006–2007 [25]. Stunting among four-year-olds declined from 10.9% to 3.6% between the 1982 and 2004 cohorts in Brazil [26]. Iriart et al. reported a stunting rate of 5.7% among Hispanic ethnic groups, compared to 1.0% among non-Hispanic White and 5.6% among all ethnicities from a U.S. sample of children 2–19 years old [27]. The prevalence of stunting fell from 32% at 1 year to plateau at approximately 3–6% from 5 years, before rising to 14–15% in boys during adolescence between 14 and 15 years of age. Stunting was significantly greater in boys than girls at 6, 14 and 15 years (*p* < 0.05, respectively). Yet, at 18%, stunting was the most prevalent indicator of undernutrition among 1–4 year old children in South Africa [28].

The highest rate of current stunting in our study of 2–8 year olds was among AIAN, suggesting concern for young child feeding practices in this group that may not promote healthy linear growth. The nutrition transition has been associated linearly with higher incomes and a transformation from an agriculture-based to a service-based economy [29], which is the situation in the USAP. The negative relationship of stunting with obesity in the USAP was unlike a study in South Africa, however [29], where the stunting levels were higher and the obesity levels were lower. In the USAP, stunting prevalence decreased with increasing country income level, and obesity increased and, in the transition, both stunting and obesity prevalences were relatively high with the dual burden of malnutrition occurring in UMIJ.

Long term adverse consequences of childhood undernutrition include impaired cognitive development, poorer educational achievement and human capital formation [3], and increased risk for obesity [4]. Thus, understanding the prevalence and patterns of undernutrition in a region in nutrition transition, such as those presented here, particularly stunting and the emergence of overweight/obesity in children and adolescents, with the concomitant risk for metabolic disease, is of critical importance for public health programming and policy.

In this study, there was more stunting present at birth than currently, although different reference data sets were used and two-thirds of the sample was missing data on length - a limitation of the study. Further the data on stunting at birth were self-reported by the caregiver, which is another limitation. Fewer missing values for birth length among the states of HI and AK (36%) than among other jurisdictions could lead to a biased subset with data. However, the data from lower income jurisdictions were likely to come from community health cards. Also, the distribution of birth length-for-age z-score in the CHL data set was reasonably symmetric (Skewness of −0.8) with a median (0.3) near 0, indicating that it does not deviate strongly for the WHO distribution. Therefore, our subset with birth length data is unlikely to be heavily biased. This report on the presence of stunting at birth in the Pacific is important as it suggests concern for fetal growth restriction and need for attention to pregnant women. This study presents a large sample of a previously unstudied in the Pacific.

Micronutrient deficits in early life also play an important role in linear growth and may result in stunting, deserving further study [30–33]. Future work should also seek measured birth length data to evaluate stunting at birth; examine maternal weight status and pregnancy weight gain; and examine infant and child dietary intake patterns, in order to further understand healthy child growth in these diverse environments. Further study of stunting and obesity is needed from birth throughout childhood, to further identify the utility of stunting as an indicator of fetal and infant undernutrition with long term consequences on obesity.

## Conclusions

Childhood stunting (at birth and at present) was highest in LMIJ. UMIJ jurisdictions had the highest dual burden of malnutrition, the highest combination of both stunting (at birth) and obesity. This illustrates that Pacific jurisdictions in economic transition have a high dual burden of malnutrition. This finding provides insight into possible targets for intervention.

## Abbreviations

AIAN: American Indian Alaska Native
CDC: Centers for disease control
CI: Confidence interval
CNMI: Commonwealth of the Northern Mariana Islands
LMIJ: Lower middle income jurisdiction
HIJ: High income jurisdiction
NHPI: Native Hawaiian Pacific Islander
OR: Odds ratio
SD: Standard deviation
SE: Standard error
UMIJ: Upper middle income jurisdiction
USAP: United States Affiliated Pacific
